# Magnesium-enhanced porcine particles using hydrothermal technique improve the osteogenic differentiation of cells†

**DOI:** 10.1039/d4ra03496a

**Published:** 2024-09-18

**Authors:** Kai-Yi Lin, Yi-Fan Wu, Lwin Moe Aung, Nai-Chia Teng, Ying-Sui Sun, Eisner Salamanca, Wei-Jen Chang

**Affiliations:** a School of Dental Technology, College of Oral Medicine, Taipei Medical University Taipei Taiwan; b Department of Biomedical Engineering, Ming-Chuan University Taoyuan Taiwan; c School of Dentistry, College of Oral Medicine, Taipei Medical University Taipei Taiwan eisnergab@tmu.edu.tw cweijen1@tmu.edu.tw +886-2-2736-2295 +886-2-2736-1661 (ext. 5150); d Department of Dentistry, Shuang Ho Hospital, Taipei Medical University New Taipei Taiwan

## Abstract

*Background*: Guided bone regeneration (GBR) uses bone grafts and barrier membranes to block soft tissue invasion and eventually create a new bone. Some studies indicate that a porcine bone graft demonstrates excellent biocompatibility and holds promise as a xenograft for GBR. However, only a few studies have investigated the effectiveness of this biomaterial after magnesium coating in improving osteoblast performance. *Aim*: This study aimed to prove that the hydrothermal method can be used to coat magnesium oxide (MgO) on the surface of a porcine graft and enhance the biomaterial's property for better osteogenic differentiation of osteoblasts *in vitro*. *Materials and Method*: A porcine bone graft was produced, and the hydrothermal method was used to coat 2 mM and 5 mM of MgO on the graft. Material physiochemistry and biocompatibility analyses were performed at days 1, 3, and 5. *Results*: pH value assay results suggested that MgO slightly increased the alkalinity of the graft. SEM images showed that MgO with some surface roughness was coated on the porcine bone surface, and EDX indicated that the Mg and O element percentages increased by about 5% and 9%, respectively. The porcine graft coated with MgO was rougher than an uncoated porcine graft. FTIR analysis of the porcine graft implied that its chemical structure did not change due to MgO hydrothermal processing. Cell viability assay illustrated the highest cell proliferation with the porcine graft with 5 mM MgO (*P* < 0.001), and good cell attachment was observed on the graft with immunofluorescence using confocal laser scanning microscopy. Cell differentiation assay results revealed that the porcine graft with 5 mM MgO had the highest alkaline phosphate activity (*P* < 0.0001) among the uncoated porcine graft and the porcine graft with 2 mM MgO. Relative quantitative polymerase chain reaction (qPCR) at days 1 and 5 revealed upregulated osteoblast gene expression with a statistically significant difference. *Conclusion*: The porcine graft hydrothermally coated with 5 mM MgO was more biocompatible and enhanced osteoblast differentiation. Thus, the findings of this study indicate that a porcine graft with 5 mM MgO has great potential as a bio-bone graft for guided bone regeneration.

## Abstract 2

Guided bone regeneration (GBR) uses bone grafts and barrier membranes to block soft tissue invasion to eventually create a new bone.

## Graphical abstract

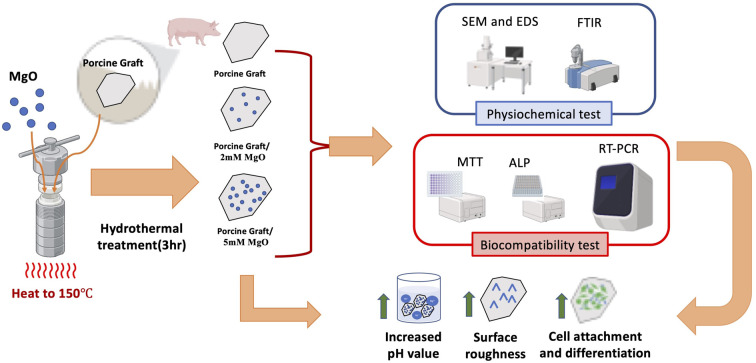

## 1. Introduction

Successful implant surgery requires adequate alveolar ridge dimensions, which are essential to house the implant and establish esthetics and function. To regenerate enough bone, guided bone regeneration (GBR) is a possible approach in many scenarios. Bone substitutes should be selected with a high demineralized bone matrix content and mechanical stability to achieve stable and successful GBR.^1^

To achieve good bone regeneration, GBR requires three elements: osteoinductive growth factors, osteoconductive matrices and osteoconductive cells. Bone grafts can be divided into autografts, allografts, xenografts, and synthetic bone grafts. Xenografts have many advantages over other grafts and are one of the most used grafts nowadays. Xenografts have major components and micro- and nanoscale structures similar to the human bone.^2,3^ Besides, xenografts have high osteoinduction and osteoconduction properties to provide a good environment for osteoblasts to attach and proliferate.^4^ More importantly, the degree of immune response to foreign substances occurring with a xenograft has been found to be smaller than that occurring with a allograft.^5^

The first choice for xenograft is usually bovine bone in dental practice. However, because of the new variant of Creutzfeldt-Jakob Disease (CJD) and v-CJD, clinicians were doubtful about using bovine bone and wanted to find different alternatives to bovine xenograft. Currently, porcine bone grafts are commonly used as substitutes for grafts and are recognized as the most similar to human bone in terms of their overall structure and microscopic composition.^6^ Comparing porcine graft to bovine graft, various studies have indicated that porcine bone has higher porosity, a large specific surface area with high surface roughness, and sub-100 nm hydroxyapatite crystals on the surface.^7^ Besides, it indicates that porcine-derived bone substitutes may offer good cell response and bone regeneration similar to commercial bovine grafts.^8^ In clinical studies, porcine xenograft also showed similar results for a sinus lift compared to the autologous bone.^9^

To further enhance the biological properties of porcine bone grafts, surface coating of the grafts with certain ions was conducted. Some ions can increase osteoblast cell proliferation, including calcium (Ca^2+^), silicon (Si^4+^), phosphorus (P^5+^), zinc (Zn^2+^), strontium (Sr^2+^), copper(ii) (Cu^2+^), boron (B^3+^), and magnesium (Mg^2+^).^10^ In recent years, magnesium ions (Mg^2+^) have been found to play an important role in bone regeneration. It induces osteogenesis, angiogenesis, and neural stimulation.^11^ Few studies have found that Mg^2+^ increases cellular adhesion through an integrin-mediated mechanism, spreading, proliferation, ALP activity, matrix mineralization, and osteogenic differentiation *in vitro* as well as enhanced osseointegration *in vivo*.^12^

Previous studies have shown that adding 5–10 mM Mg^2+^ enhances the mineralization of ECM in magnesium alloy.^13^ Another study demonstrated that in the presence of 2 mM, Mg^2+^ enhanced the growth and specialization of pre-osteoblasts and increased the expression of genes related to bone formation. However, a concentration of 5 mM Mg^2+^ had a detrimental effect on osteoblast specialization and the metabolic processes involved in bone formation, potentially leading to deficiencies in bone mineralization.^14^ Therefore, in this study, we attempt to create a new biomaterial using magnesium oxide (MgO) coated on a porcine graft surface and its capability for bone tissue engineering treatment.

To enhance the bioactive properties of the porcine graft by coating MgO on its surface and to achieve optimal biological properties, the hydrothermal method was utilized. The hydrothermal method can grow various single crystals to prepare less agglomerated crystallized ceramic material at a relatively low temperature.^15^ Besides, it can produce materials that exhibit low stability at high temperatures and have higher vapor pressures, resulting in minimum material loss. Furthermore, it can be regulated by liquid phase or multiphase chemical reactions.^16^ This Mg-doping method has been proven previously by adding 1 wt% MgO to HA/β-TCP ceramic and drastically increasing its mechanical properties compared to the HA or β-TCP ceramics alone, without changing the biological safety and biocompatibility of the original composite.^17^ Studies have proven that the hydrothermal addition of MgO to the β-TCP surface can increase biocompatibility and roughness to enhance cell viability and proliferation without changing the elemental composition of the bone particles.^18^ This study aimed to prove that the hydrothermal method can coat magnesium oxide (MgO) on the surface of the porcine graft and enhance biomaterial properties for better osteoblast's differentiations and osteogenic properties.

## 2. Materials and methods

### 2.1 Modified porcine graft preparation

#### 2.1.1 Porcine graft manufacturing

**Fig. 1:**
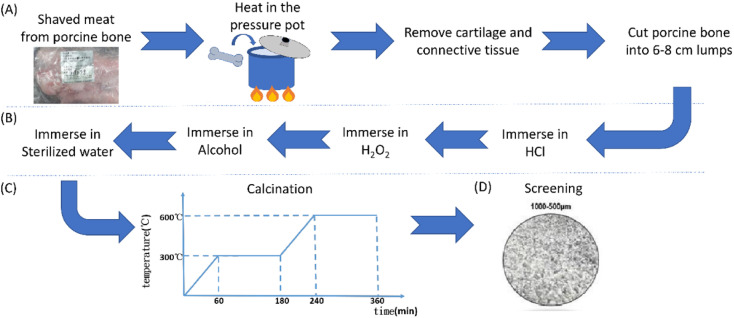
Protocol for a porcine bone graft. (A) Pre-treatment, (B) acid treatment, (C) calcination, and (D) the finished porcine graft.

Based on previous optimal findings,^6^ porcine grafts were produced with a better particle size of 500–1000 μm. This experiment used biomedical porcine bone processed in three steps: pre-treatment, acid treatment, and calcination. In pre-treatment, we must shave residual meat on the porcine bone and put processed porcine bone in a pressure pot at 400 °C for 4 hours to remove oil from the porcine bone. Then, porcine bone easily eliminated cartilage and connective tissue after heating, and it was continuously heated in a pressure pot at 500 °C for 120 hours. Continuously, we cut porcine bone into 6–8 cm lumps with a wire saw. After pre-treatment, these lumps are processed with acid treatment. Immersing porcine bone in 0.5 N hydrochloric acid (HCl) for 24 hours, we immersed porcine bone in 3.5% hydrogen peroxide (H_2_O_2_) for 30 min and 75% alcohol for one hour. Then, we bathe lumps in sterilized water for 72 hours and put them in the oven to dry. After finishing the acid treatment, we calcinate these lumps using a muffle furnace, and the temperature–time associated figure is shown in Fig. 1. Finally, we make these lumps become different particle sizes and screen the particle sizes to 500–1000 μm.

#### 2.1.2 Hydrothermal method

The hydrothermal method is used to create the environment in 100–1000 °C and 1–100 MPa, making insoluble matter dissolve and recrystallize. In this experiment, we mainly needed magnesium oxide (MgO), sodium hydroxide (NaOH_(aq)_), and porcine graft. A 40 mL solution containing 0.2 M NaOH_(aq)_ and either 0, 2, or 5 mM MgO was prepared. This solution and porcine graft are placed in the hydrothermal container and sealed. The container is heated to 150 °C for 3 hours. Finally, the solution is filtered, and a modified porcine graft-covered MgO coating is obtained. Besides, reserve materials in the oven at 60 °C.

### 2.2 Material characterization

#### 2.2.1 pH value assay

The electrode is placed in the solution to be tested. If the hydrogen ion concentration of the solution to be tested is different, the electrode changes the potential, and the pH value of the solution to be tested can be obtained by the linear relationship between the potential and the pH value. Before the pH value assay, we need to put the porcine graft in sterilized water.

#### 2.2.2 Scanning electron microscopy (SEM) and energy dispersive spectrometry (EDS)

A layer of gold film was coated on the surfaces of the porcine graft specimens (test group sample with different concentrations of MgO, *n* = 4). The observed surface area of different porcine grafts was chosen for photographs, which operated at 15 kV with 60×, 1500×, and 2,500× magnification. Images were taken from at least five random, nonoverlapping flat areas. Microphotography was taken using a scanning electron microscope (SEM S-2400; Hitachi, Ltd, Tokyo, Japan), and the composition of the element analysis was determined using energy dispersive spectroscopy (EDS, Bruker Quantax EDS, Germany).

#### 2.2.3 Image processing

After completing the scanning electron microscopy, we used ImageJ to verify the roughness of the porcine graft surface precisely. The surface roughness is assessed, and the data are processed from scanning electron microscope (SEM) images at a magnification of 500×. By utilizing a scale bar, the precise dimensions of the object can be accurately documented in the photograph. If the entire area of the shot is cropped, the level of roughness in that area is observed in the picture. Besides, SurfcharJ was used to show the data on arithmetic mean roughness (*R*_a_), total roughness (*R*_t_), mean height (*R*_c_), and square roughness (*R*_q_) values of porcine bone surface roughness. *R*_a_ is the average of the absolute value along the peak or valley height. *R*_q_ is the geometric mean of the peak or valley height. *R*_t_ was the maximum total peak and valley height. *R*_c_ is the mean for adding the peak and valley height of each profile element.

#### 2.2.4 Functional group analysis using ATR-FTIR

ATR-FTIR analysis of the modified graft samples was performed using a Nicolet iS5 (Thermo Fisher Scientific, Madison, WI, USA) equipped with an iD7 crystal ZnSe in reflection mode. The absorbance spectra of the control and fibronectin-grafted samples were measured using 16 scans with a resolution of 0.482 cm^−1^. FITR spectra were obtained in the wavenumber ranging from 4000 to 650 cm^−1^, and a background spectrum was used to normalize the spectra. The absorbance of the spectra is measured to derive atomic peaks.

### 2.3 Biocompatibility evaluation

#### 2.3.1 Cell culture and seeding

MG-63 human osteoblast-like cells were purchased from the Bioresource Collection and Research Center (BCRC, Hsinchu, Taiwan). Following a published protocol, MG-63 cells were cultured in Dulbecco's Modified Eagle's Medium (DMEM; HyClone, Logan, UT, USA) supplemented with l-glutamine (4 mmol L^−1^), 10% fetal bovine serum (DMEM; HyClone, Logan, UT, USA) at 37 °C in a humidified atmosphere serum, and 1% penicillin–streptomycin (HyClone, Logan, UT, USA) at 37 °C in a humidified atmosphere containing 95% air and 5% CO_2_. Upon reaching approximately 80% confluence, the cell concentration was adjusted to 5 × 10^4^ cells per well and seeded into 24-well Petri dishes (Costar Corporation, Cambridge, MA, USA) or 1 × 10^4^ cells per well and seeded into 96-well Petri dishes (Costar Corporation, Cambridge, MA, USA) for subsequent experiments.

#### 2.3.2 Cell viability

Cell viability was assessed using a 3-(4,5-dimethylthiazol-2-yl)-2,5-diphenyltetrazolium bromide (MTT) reduction assay (Roche Applied Science, Mannheim, Germany). The MG-63 cells were seeded into a 24-well Petri dish and waited 24 hours for the cells to attach to the dishes. Then, the original medium is replaced with the medium cultured collectively with porcine graft for 24 hours. Formazan was generated in viable cells by mitochondrial dehydrogenase following the addition of MTT salt, as per the directions provided by the manufacturer. The formazan dye was dissolved in dimethyl sulfoxide (DMSO) for 10 minutes, resulting in a transformation in hue from yellow to a deep blue shade. Subsequently, the optical density of the medium was quantified using an ELISA reader (SpectraMax iD3 Multi-Mode Detection Platform, Molecular Devices, USA) at 540 nm. Cell viability was expressed as a percentage, assigning the 100% optical density value to the absorbance of the control cells. In this study, each group was repeated at least 3 times and evaluated on days 1, 3, and 5.

#### 2.3.3 Immunofluorescence

MG-63 cells were cultured with porcine bone graft and medium. After 24 hours of MG-63 cell growth, all samples were removed from their medium, rinsed with PBS (10 mM, pH 7.4), and fixed with 4% paraformaldehyde (PFA) at room temperature. The cells were permeabilized with 1% Triton X-100 in PBS for 10 minutes at room temperature. After washing three times with 0.1% Triton X-100 in DPBS, the nuclei were stained for 1 h with DAPI (1 : 1000 dilution; 5 mg mL^−1^ stock solution; Sigma-Aldrich) and Alexa Fluor 488 Phalloidin (1 : 80 dilution; catalog # A12379; Invitrogen). PBS was used to eliminate excess coloring (10 mM, pH 7.4).^19^ Leica STELLARIS 8 systems were utilized to evaluate the dispersion of cells throughout the various porcine graft samples.

#### 2.3.4 Alkaline phosphatase (ALP) activity

ALP level was determined in MG-63 (1 × 10^4^ cells per well in 96-well Petri dishes). After cell attachment, the original medium was replaced with the medium cultured with porcine graft for 24 hours. According to the manufacturer's protocol (Abcam, ALP kit catalog #ab83371, California, USA), intracellular ALP measurement was performed by putting the culture supernatants in 50 μL of assay buffer in a 96-well plate (Costar Corp., Cambridge, MA, USA). Subsequently, 20 μL of stop solution and 50 μL of 5 mM 4-nitrophenyl phosphate (pNPP, Merck, Schuchardt, Hohenbrunn bei München, Germany) were added to each well. After pipetting and waiting for hydrolyzing by ALP for 60 minutes at 25 °C, a yellow product (nitrophenol) was formed later. An enzyme-linked immunosorbent assay reader (SpectraMax iD3 Multi-Mode Detection Platform, Molecular Devices, USA) was used to evaluate the absorbance, which was measured at a wavelength of 405 nm. The activity of ALP (unit per L) was determined.

#### 2.3.5 Real-time polymerase chain reaction

MG-63 cells were cultured with porcine bone graft and medium, with 1 × 10^5^ cells per well in 6-well plates. Quantification of all gene transcripts was performed on days 1 and 5 after cell culture and seeding (control and test group samples with different concentrations of MgO, *n* = 4). Total RNA extraction and purification were carried out using the Novel Total RNA Mini Kit (NovelGene, Molecular Biotech, Taiwan) according to the manufacturer's instructions. For RNA processing, cells were exposed to 1 mL Trizol and lysed by adding 200 μL chloroform and 500 μL isopropanol to the sample. RNA binding was later performed with 400 μL of 70% ethanol and centrifuged at 12 000 rpm. Afterward, the sample was washed and eluted with 20 μL of RNase-free water. Next, purified RNA was quantified using an ND-1000 spectrophotometer (Nanodrop Technology, Wilmington, DE, USA). Each sample was stored at −20 °C for the analysis of the real-time polymerase chain reaction (qPCR).

The expression levels of osteoblast markers, including Distal-Less Homeobox 5 (DLX5), osteocalcin (OC), runt-related transcription factor 2 (RUNX2), transcription factor Osterix (SP7), alkaline phosphatase (ALP), osteoprotegerin (OPG), and the receptor activator of nuclear factor kappa B (RANK), were quantified using qPCR. Gene expression levels were normalized to the expression of the housekeeping gene glyceraldehyde 3-phosphate dehydrogenase (GAPDH). The control cell's genes were set as the calibrator sample in the DEME medium, representing the transcript amount expressed on day 0 of cells cultured only in the DMEM medium.

Real-time PCR was performed using 1 μL of cDNA; 9 μL of reaction volume with a LightCycler® 96 Instrument; an application software (Roche Molecular Systems, Inc., California USA); and the Fast SYBR™ Green Master Mix (Thermo Fisher, Cat#4344463, Madison, WI, USA). The temperature profile of the reaction was 95 °C for 10 min, followed by 40 cycles of denaturation at 95 °C for 15 s and annealing at 60 °C for 30 s. Quantification was performed using the delta–delta calculation method. The cycle threshold (*C*_T_) value was used as an indicator, and the gene expression levels were normalized using GAPDH levels in each sample to account for differences in the total RNA content in the individual samples. Forward and reverse primer sequences were designed using Primer-BLAST from the U.S. National Library of Medicine.

## 3. Results

### 3.1 pH assay

The results of the pH assay showed that the MgO coating on the porcine graft was directly proportional to a moderately more alkaline pH, with porcine graft/5 mM MgO at days 1 and 5, and the biomaterial with the highest basic pH levels at 8.6 and 8.7, respectively. The results demonstrate that the pH value of the porcine graft with 2 mM MgO and 5 mM MgO shifted from 7.5 to 7.6, 8.1 to 8.3 and 8.6 to 8.7, respectively, from day 1 to 5. The sterilized water was measured at 6.76.

### 3.2 Surface morphology observation and energy dispersive spectrometry testing

**Fig. 2:**
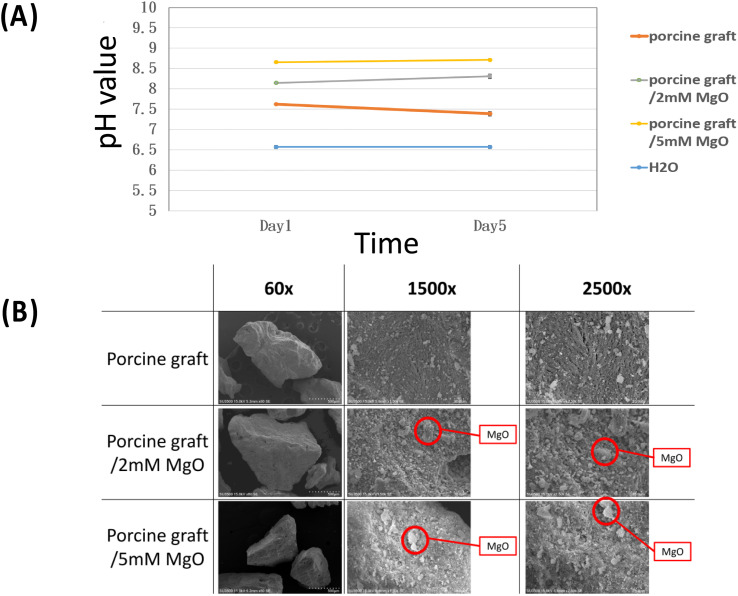
(A) pH value assay indicating a porcine bone and porcine graft with 2 mM MgO and 5 mM MgO more alkaline pH than control. (B) SEM images of different porcine graft samples exhibiting the roughness difference between porcine bone particles, porcine bone coated 2 mM magnesium oxide and porcine bone coated 5 mM magnesium oxide (1500× and 2500×).

The surface morphology at the macro scale (60X images) displayed similar particle size and patterns in all porcine graft samples (Fig. 2B). On a micro scale, porcine bone, porcine graft/2 mM MgO and porcine graft/5 mM MgO rough surfaces were observed (1500×, 2500× Fig. 2B).

Quantitative topographical evaluations conducted by EDX revealed that all samples contained oxygen, phosphorus, calcium, magnesium, and carbon (Table 1). The weight percentages of oxygen, phosphorus, calcium, magnesium, and carbon in porcine bone were 32.15 ± 6.03%, 12.66 ± 2.72%, 30.20 ± 8.70%, 016 ± 0.02%, and 4.54 ± 0.62%, respectively. The weight percentage of these elements of porcine bone weights increased in the porcine bone/2 mM MgO samples at 1.1 ± 0.26% magnesium and 37.74 ± 7.37% oxygen and in the porcine bone/5 mM MgO samples at 4.92 ± 1.53% magnesium and 41.38 ± 2.21% oxygen, due to magnesium oxide coating on the surfaces (Table 1). The Ca/P ratio was 2.39 in the porcine graft. Porcine graft/2 mM MgO was approximately 4.43, and porcine graft/5 mM MgO was 2.12.

**Table 1:** Weight ratio percentages of porcine graft samples

Chemical element	Porcine graft (wt%)	Porcine graft/2 mM MgO (wt%)	Porcine graft/5 mM MgO (wt%)
Mg	0.16 ± 0.02	1.1 ± 0.26	4.92 ± 1.53
Ca	30.20 ± 8.70	10.37 ± 1.94	15.71 ± 2.45
P	12.66 ± 2.72	2.34 ± 0.3	7.4 ± 2.09
O	32.15 ± 6.03	37.74 ± 7.37	41.38 ± 2.21
C	4.54 ± 0.62	4.43 ± 1.07	7.18 ± 0.25

### 3.3 Surface roughness analysis

**Fig. 3:**
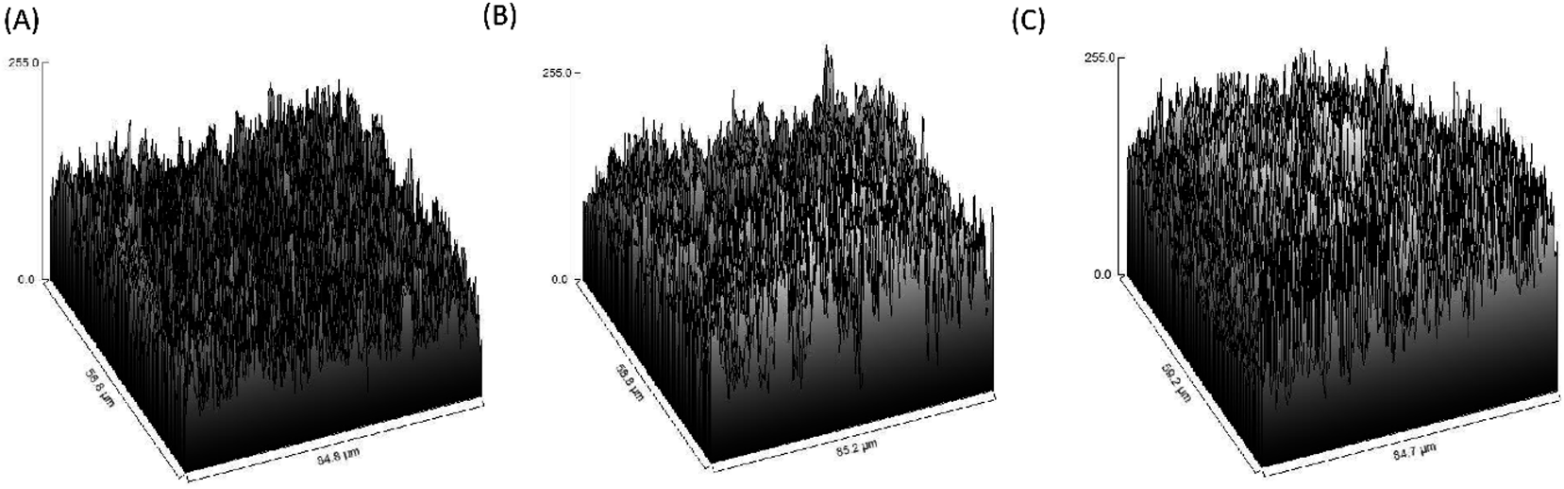
Roughness level of the figures was processed using 500× magnification SEM images: (A) porcine graft, (B) porcine graft/2 mM MgO and (C) porcine graft/5 mM MgO.

The image differed greatly from the porcine graft/MgO and porcine graft. The surface of porcine bone particles after coating on magnesium oxide was rougher than pure porcine bone, as shown in Fig. 3. Accordingly, SurfCharJ was used to determine arithmetic mean roughness (*R*_a_), total roughness (*R*_t_), mean height (*R*_c_), and square roughness (*R*_q_) values of porcine bone surface roughness (Table 2). In particular, *R*_a_ and *R*_q_ were widely used as parameters for roughness. The *R*_a_ and *R*_q_ values of the porcine graft were 18.0928 and 24.2577 μm, respectively. The *R*_a_ and *R*_q_ values of porcine graft/2 mM MgO were 28.2031 and 35.5426 μm, respectively. The *R*_a_ and *R*_q_ values of porcine graft/5 mM MgO were 40.3499 and 49.7454 μm, respectively. Accordingly, the *R*_t_ and *R*_c_ values were also recorded (Table 2), indicating that porcine graft/5 mM MgO had the highest surface roughness.

**Table 2:** The difference between the uncoated and coated porcine bone grafts with respect to average roughness (*R*_a_), square roughness (*R*_q_), total roughness and mean height (*R*_c_)

	*R*_q_	*R*_a_	*R*_t_	*R*_c_
Porcine graft	24.2577	18.0928	204.8304	−1.016
Porcine graft/2 mM MgO	35.5426	28.2031	245.3039	0
Porcine graft/5 mM MgO	49.7454	40.3499	265.2163	2.1405

### 3.4 Functional group results by ATR-FTIR

**Fig. 4:**
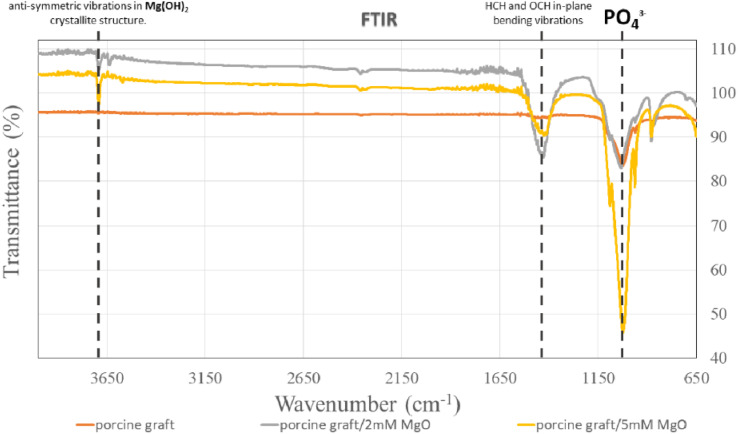
Analysis of atomic peaks and functional groups *via* infrared spectroscopy (FTIR) spectra.

Phosphate (PO_4_^3−^) was shown at an absorption band of 1028 cm^−1^ in all the groups (Fig. 4). For 2 mM and 5 mM groups, it could also be observed that there were similar peaks at 1430 cm^−1^ illustrating HCH and OCH in-plane bending vibrations and 3694 cm^−1^ for anti-symmetric vibrations in Mg(OH)_2_ crystallite structure.

### 3.5 Cell viability and proliferation

**Fig. 5:**
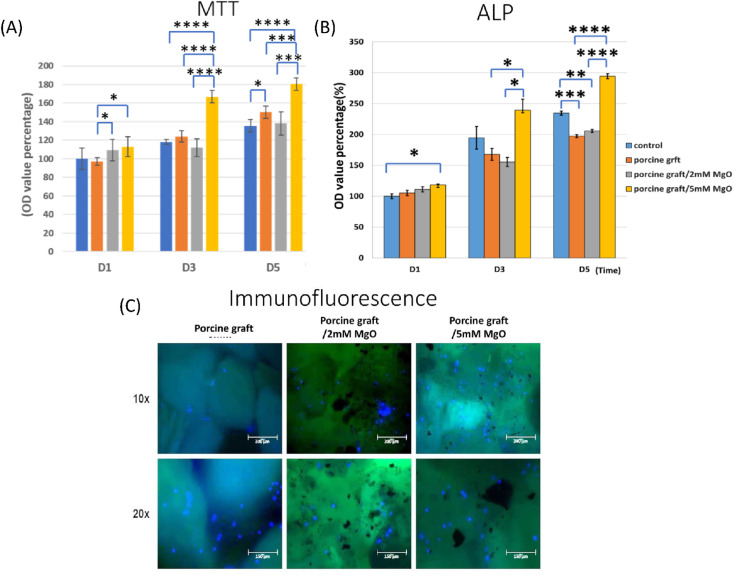
(A) Proliferation determined *via* MTT cell assay of MG-63 on different porcine graft samples. *P < 0.05, significant difference; **P < 0.01, ***P < 0.001; ****P < 0.0001, significant difference. (C) 24 hours cell culture with DAPI/phalloidin immunofluorescent imaging, indicating that the MG-63 cell is attached on all porcine graft surfaces. (B) Alkaline phosphate activity detection was shown by all porcine graft biomaterials, with all the porcine graft exhibiting a slightly higher percentage than other groups.

The MTT assay (Fig. 5A) and 3D cell culture immunofluorescence assay (DAPI–phalloidin) (Fig. 5B) were utilized to determine cell viability and proliferation. Porcine bone 5 mM MgO had the highest cell proliferation, especially on day 3 and day 5, with 162.29% and 180.32% over control, respectively. Besides, on day 5, the figure presented the statistically significant difference between porcine bone 5 mM MgO and all the other groups. Phalloidin/DAPI immunofluorescence staining demonstrated that MG-63 confirmed the cells attached to the porcine graft surface using confocal laser scanning microscopy.

### 3.6 Cell differentiation activity

Alkaline phosphatase enzyme activity detection showed results with a significant difference, especially for porcine graft with 5 mM MgO samples at 5 days (Fig. 5C). Porcine graft with 5 mM MgO on day 3 increased to 239.4, and the 2 mM MgO-coated porcine graft was lower than that in this group. Porcine graft with 5 mM MgO had a better ALP percentage of 294.4 compared to the other groups and resembled ALP gene expression results at day 5. Interestingly, the 2 mM MgO-coated porcine graft on day 5 indicated that it was 10 percent better than the porcine graft alone.

### 3.7 Real-time polymerase chain reaction

**Fig. 6:**
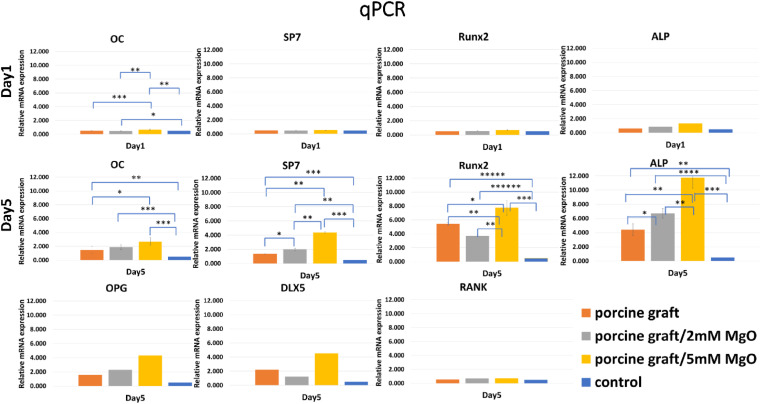
mRNA expression of osteogenesis-related genes. Porcine graft/5 mM MgO samples showed the highest gene expression. *P < 0.05, **P < 0.01, ***P < 0.001, ****P < 0.001 significant differences.

Osteogenesis-related genes, such as OPG, DLX5, and RANK, were not expressed on day 1 but showed upregulation in porcine graft on day 5. OC is secreted by osteoblast during extracellular matrix mineralization. The results demonstrated that the OC biomarker on day 5 increased slightly compared to that on day 1. The expression of immature osteoblast makers, SP7 and Runx2, was presented slightly on day 1. Then, both increased exponentially in cells cultured with all the porcine grafts, with 7.757 in the 5 mM MgO/porcine graft. The positive regulator gene for osteoblastogenesis, DLX5, was absent on day 1, but it was later expressed on day 5. The expression of the osteoclast marker, RANK, was upregulated on day 5. The osteoclast differentiation factor, OPG, had 4.317, the highest expression, in the cell culture with porcine graft coated with 5 mM MgO. ALP, the early marker of osteoblast differentiation, was expressed similarly to that in the ALP assay results (Fig. 5). Besides, cells cultured in all the porcine graft groups showed significant differences for osteoblast-related genes, especially on day 5. Overall, cells cultured with porcine graft coated with 5 mM MgO demonstrated the best expression of genes related to osteoblast-like cell osteogenesis (Fig. 6).

## 4. Discussion

Porcine bone grafts are used for guided bone regeneration in horizontal and vertical bone regeneration, ridge augmentation, sinus grafting,^20^ and bone grafting for implant placement^21^ due to their beneficial osteoconductivity and biocompatibility.^22,23^ Porcine bone graft has proven to be a unique clinical material for guided bone regeneration that does not interfere with wound healing and promotes osteoconduction bone formation.^24^ It also had similar and comparable efficacy to other commercial bone grafts, such as HA/β-TCP.^6^ Although bone regeneration procedures have good qualities, they can still be improved to increase bone formation. In this study, our novelty is to modify porcine graft by coating magnesium oxide with hydrothermal addition on its surface to strengthen this biomaterial surface and could become more biocompatible than porcine bone graft alone. Therefore, to accomplish our goal, we utilized a hydrothermal approach to apply a coating of MgO onto the surfaces of porcine bone graft particles. The study demonstrated that hydrothermal treatment was a highly efficient technique for using a layer of MgO on the surface of porcine bone particles (Fig. 1 and 2). Compared to other coating methods, hydrothermal treatment is cost-effective and more eco-friendly.^25^ In our study, hydrothermal treatment required only one reaction without the need for additional heat treatment, as previous studies have reported.^26^

Magnesium ions at 1 wt% have undoubtedly been proven to improve biological apatite by upregulating osteogenic genes and bone formation, helping to regulate changes in the bone matrix, indirectly influencing mineral metabolism, modifying catalytic reactions, and controlling biological functions.^17^ The MgO coating facilitates a localized release of magnesium ions. This release creates a mild alkaline microenvironment. This optimized pH condition is associated with enhanced osteoblast activity, promoting more effective bone regeneration.^27^ This induced activity of osteoblasts is encouraged by enhancing gap junction intercellular communication (GJIC) between cells and promoting bone formation.^28^ In the present study, the addition of 2 mM MgO to porcine particles resulted in a 1.1 ± 0.26 wt% concentration and an alkaline pH value of 8.3. In comparison, the addition of 5 mM MgO achieved a higher concentration of 4.92 ± 1.53 wt% and a more alkaline pH value of 8.7. The 5 mM MgO coating significantly enhanced cell bioactivity and osteoblast differentiation compared to both the porcine control and the porcine graft/2 mM MgO group (*P* < 0.001, as shown in Fig. 5A, B and 6). The porcine graft with 5 mM MgO exhibited a more significant overexpression of osteogenic genes compared to the porcine graft and porcine graft with 2 mM MgO, with a statistically significant difference (Fig. 6).

Another study optimized the properties of spherical granules of hydroxyapatite bone substitutes sintered with MgO (HAp/MgO), resulting in better cell osteoblast proliferation.^29^ Similar viability was found in cells cultured with porcine graft/2 mM MgO and porcine graft/5 mM MgO. In contrast to the findings reported in the literature, different poly(l-lactic acid),^30^ nanobioglass,^31^ and HA/β-TCP biografts^32^ containing MgO were utilized to conduct cytotoxicity experiments. Various concentrations of MgO applied to the biografts enhanced cell viability. Nevertheless, few studies have explored the osteogenic capabilities of porcine bone graft when paired with a biologically significant mineral element, such as magnesium (Mg).

The SEM surface morphology showed the addition of MgO to the porcine graft surface without altering the structure of the particles (Fig. 2), which increased the pH of the graft material (Fig. 2). EDX revealed that the element weight percentages of magnesium and oxide increased (Table 1), supporting the results that magnesium oxide was coated on the porcine bone surface. Fig. 3 and Table 2 demonstrate that the magnesium oxide-coated porcine graft was statistically significantly rougher than the porcine graft, as indicated by mean roughness (*R*_a_), square roughness (*R*_q_), total roughness (*R*_t_), and mean height (*R*_c_). Specifically, mean roughness and square roughness were selected as metrics that accurately represent the roughness of the surface. Therefore, these findings demonstrate that the porcine graft becomes rougher with a quantitative increase when magnesium oxide is applied to its surface. There have been studies indicating that bone grafts with higher roughness promote lower cytotoxicity levels and better biocompatibility to cell response.^33^ This indicates that porcine graft/5 mM MgO exhibits greater osteoblast differentiation and cell viability as was seen in MTT, ALP, and qPCR results at day 5 (Fig. 5A, B and 6).

All porcine grafts had similar FTIR spectra (Fig. 4), indicating that the hydrothermal method did not change the functional group and structure of the sample materials. However, the results demonstrated slight differences in the structure of the porcine graft/2 mM MgO and porcine graft/5 mM MgO. The results showed that both porcine grafts, after being coated with magnesium oxide using the hydrothermal method, had peaks at 1430 and 3693 cm^−1^. Previous studies have demonstrated that a 3693 cm^−1^ peak resulted from anti-symmetric stretching vibrations in the magnesium hydroxide crystallite structure.^34^ Additionally, the peak at 1430 cm^−1^ was the HCH and OCH in-plane bending vibrations. Moreover, the absorbance ratio of the bands at 1430 and 987–893 cm was closely related to the portion of the cellulose I structure.^35^

Previous studies compared different particle sizes and found that 500–1000 μm was better than 250–500 μm particles for bone regeneration. In this study, the 500–1000 μm porcine graft was proven to exhibit the greatest biocompatibility.^6^ Besides, deproteinized bovine bone mineral (DBBM) and deproteinized porcine bone mineral (DPBM) can comparably augment damaged extraction sockets with minimal postoperative reduction of the grafted volume.^36^ In another study, MG-63 cells were attached to the surface of magnesium-coated β-TCP for 24 hours.^18^ It was similar to the cell culture with a DAPI/phalloidin immunofluorescent image (Fig. 5C).

Overall, cells cultivated with porcine graft hydrothermally treated with 5 mM MgO (Fig. 6) had the best expression of genes related to osteoblast genesis and promoted osteogenic proliferation and differentiation of MG-63 cells *in vitro*. However, this *in vitro* study still has some limitations to prove further improved osseointegration properties. Therefore, its detailed mechanism on new bone formation should be explored further, and the *in vitro* data obtained in this study should be confirmed by continuing research in animal studies to ensure that this improved porcine graft can be employed in clinical practice.

## 5. Conclusions

According to our research findings, porcine grafts coated hydrothermally with 5 mM MgO exhibited the most favorable physicochemical and biological properties. This grafts showed the superior performance in terms of osteoblast proliferation, differentiation, and osteogenic differentiation *in vitro*. This hydrothermal treatment method for porcine bone grafts coated with magnesium demonstrated optimal properties and proved its potential for future use. After investigating its properties *in vivo*, it can be considered an alternative bone graft for future guided bone regeneration in dental treatments.

## Data availability

Data is contained within the article and ESI.†

## Author contributions

Y.-F. W and W.-J. C conceived and designed the experiments; K. Y. L, Y.-F. W, L.-M. A, and E. S. performed the experiments; K. Y. L, Y.-F. W, and E. S. analyzed the data; K. Y. L, Y.-F. W, L.-M. A, N.-C. T, Y.-S. S, E. S and W.-J. C. wrote and review the manuscript; K. Y. L and E. S. received the funding about this research.

## Conflicts of interest

The authors declare that there are no conflicts of interest regarding the publication of this paper.

## Supplementary Material

RA-014-D4RA03496A-s001
