# Supplementary material for: Magnesium-enhanced porcine particles using hydrothermal technique improve the osteogenic differentiation of cells
Source: RSC Adv. 2024 Sep 18;14(40):29455–63. doi: 10.1039/d4ra03496a (PMC11409227; doi:10.1039/d4ra03496a)
Supplement: RA-014-D4RA03496A-s001 [file RA-014-D4RA03496A-s001.pdf]

Supplementary Information (SI) for RSC Advances.
This journal is © The Royal Society of Chemistry 2024

Supplementary Information

The manufacture of porcine graft preparation

1.1. Pre-treatment

1.1.1. Porcine bone for biomedical (bought from Agricultural Technology Research Institute, Fig. S1A) was put into a pressure pot and distilled water until the volume ratio for porcine bone and water was 1:20.

1.1.2. Use the DLAB hot plate stirrer (DLAB MS7-H550-S). Set temperature at 400°C for heating for 4 hours to make oil release.

1.1.3. After removing cartilage, connective tissue, and pork, set the temperature at 500°C for heating for 120 hours. (Fig. S1B)

1.1.4. A jigsaw cut the Porcine bone in lumps about 6-8 centimeters. (Fig. S1C)

Fig. S1. (A) Porcine bone for biomedical (B) Porcine bone after heated (C) Cut porcine bone

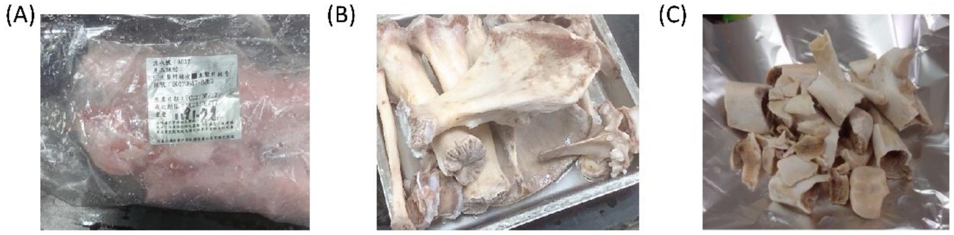

1.2. Acid treatment (Fig. S2A)

1.2.1. Immerse porcine bone lumps in hydrochloric acid (0.5N HCl) for 30 minutes to achieve deproteinization, and rinse porcine bone lumps.

1.2.2. Put the porcine bone in hydrogen peroxide (3% H_2O_2) for 30 minutes and wash porcine bone.

1.2.3. Dip porcine bone in 75% alcohol for 1 hour.

1.2.4. Use double distilled water(ddH2O) to rinse the porcine graft for 12 hours, and repeat this step, until ddH2O is clear.

1.2.5. Dry porcine graft at 60°C in the oven (DK-500DT).

1.3. Calcination (Fig. S2B)

1.3.1. Use a high-temperature furnace and set program according to Fig.1c

1.4. Screening porcine graft to 500-1000μm.

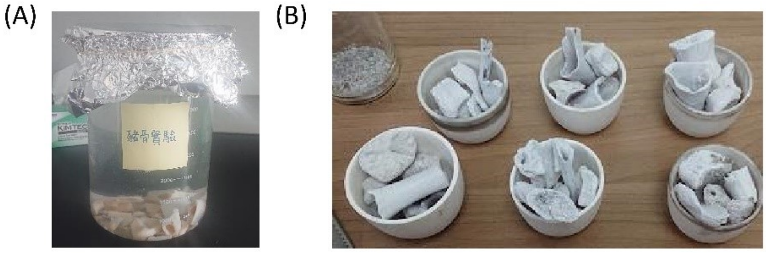

Fig. S2. (A) Porcine bone in acid treatment (B) Porcine bone after calaination

2. Hydrothermal treatment

   2.1. Add 40ml 0.2M sodium hydroxide (NaOH) and put in 50mg/125mg MgO (2mM/5mM) in hydrothermal container.

   2.2. Put porcine graft in a container, stir, and seal the container

   2.3. Heat at 150°C for 2 hours, and filter solution.

   2.4 Dry sample at 60°C in the oven.
